# THR Simulator – the software for generating radiographs of THR prosthesis

**DOI:** 10.1186/1471-2474-10-8

**Published:** 2009-01-16

**Authors:** Tai-Yin Wu, Rong-Sen Yang, Chiou-Shann Fuh, Sheng-Mou Hou, Chen-Kun Liaw

**Affiliations:** 1 Taipei City Hospital, Renai Branch, Tapei City, Taiwan; 2 Department of Orthopaedics, College of Medicine, National Taiwan University & Hospital, Tapei City, Taiwan; 3 Institute of Computer Science and Information Engineering, National Taiwan University, Tapei City, Taiwan; 4 Department of Orthopaedics, Tao-Yuan General Hospital, Tao-Yuan, Taiwan; 5 Minghsin University of Science and Technology, Hsinchu County, Taiwan; 6 Ming Chuan University, Tapei City, Taiwan

## Abstract

**Background:**

Measuring the orientation of acetabular cup after total hip arthroplasty is important for prognosis. The verification of these measurement methods will be easier and more feasible if we can synthesize prosthesis radiographs in each simulated condition. One reported method used an expensive mechanical device with an indeterminable precision. We thus develop a program, *THR Simulator*, to directly synthesize digital radiographs of prostheses for further analysis.

Under Windows platform and using Borland C++ Builder programming tool, we developed the *THR Simulator*. We first built a mathematical model of acetabulum and femoral head. The data of the real dimension of prosthesis was adopted to generate the radiograph of hip prosthesis. Then with the ray tracing algorithm, we calculated the thickness each X-ray beam passed, and then transformed to grey scale by mapping function which was derived by fitting the exponential function from the phantom image. Finally we could generate a simulated radiograph for further analysis.

**Results:**

Using *THR Simulator*, the users can incorporate many parameters together for radiograph synthesis. These parameters include thickness, film size, tube distance, film distance, anteversion, abduction, upper wear, medial wear, and posterior wear. These parameters are adequate for any radiographic measurement research. This *THR Simulator *has been used in two studies, and the errors are within 2° for anteversion and 0.2 mm for wearing measurement.

**Conclusion:**

We design a program, *THR Simulator *that can synthesize prosthesis radiographs. Such a program can be applied in future studies for further analysis and validation of measurement of various parameters of pelvis after total hip arthroplasty.

## Background

Measuring the orientation of acetabulum cup and the wearing of insert on plain radiograph of patients who underwent total hip arthroplasty is important for prognosis[1]. Verifying the orientation measurement [1-6] and wearing [7-14] methods are both important, which may require a simulator to mimic every situation for such an analysis[1]. Mechanical simulator has once been reported in a study to measure the wearing of acetabular insert [7]. Although such a mechanical device is straightforward, there are disadvantages including expensive price, undetermined precision, as well as requiring image processing from radiograph to digital form. Every processing step may cause error and interfere with the final precision.

**Figure 1:**
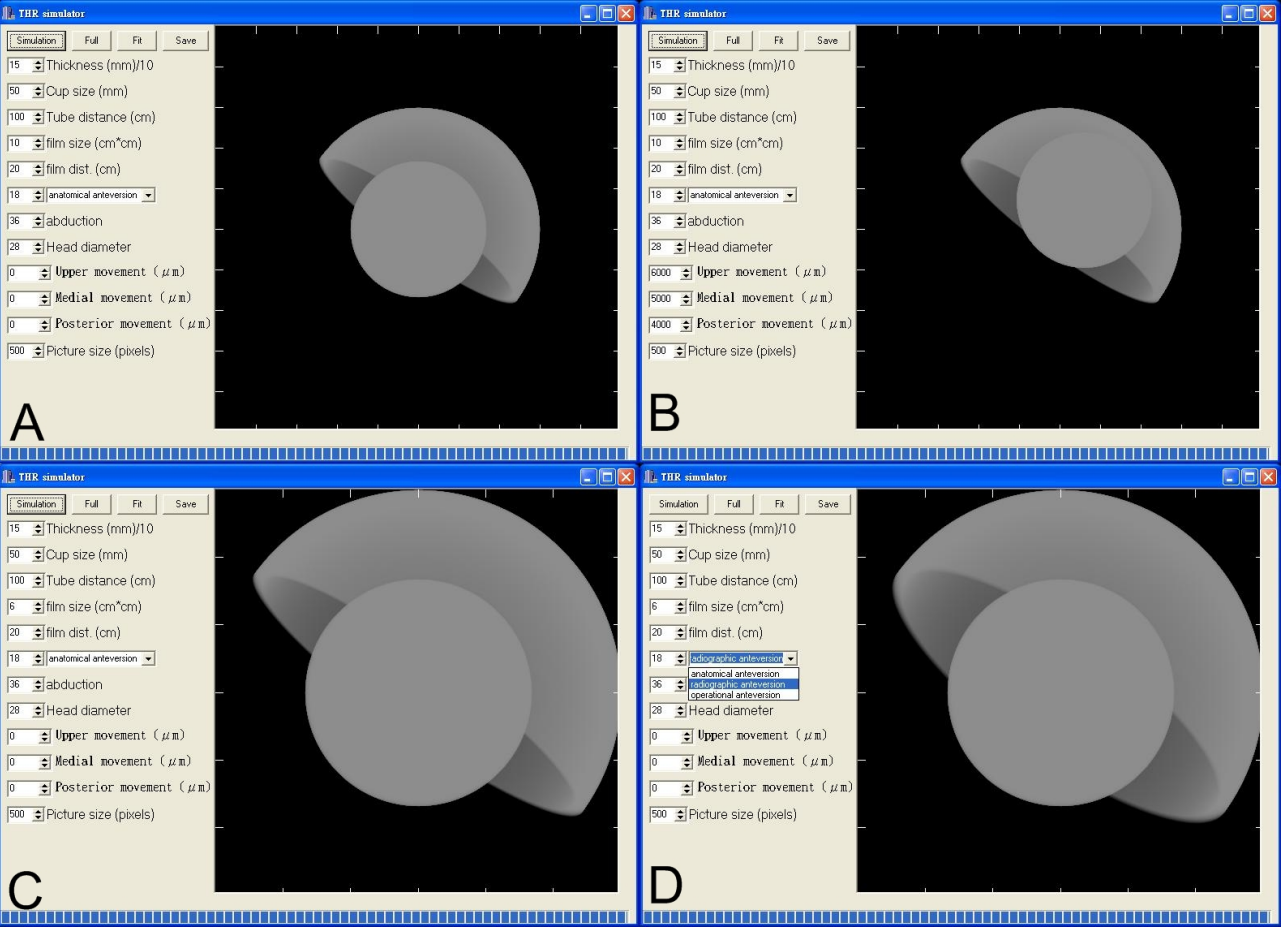
**(A) The *THR Simulator *and the basic figure of the generated radiograph**. (B)We adjusted the wearing parameters and the result showed that the femoral head is centrally migrated. (C)We adjusted the film size parameter to simulate a smaller film in the same picture and the result is shown. (D)We chose the anteversion type as radiographic anteversion and the result is shown.

We thus developed our own *THR Simulator *[Figure 1] by designing a software program that can be used to generate digital radiographs directly. The early edition of *THR Simulator *software has been used to simulate 45 radiographs of total hip arthroplasties with 15 different anteversions ranging from 15°–29°, and then to verify the protractor which was designed to measure the anteversion of acetabular cup [1]. We have corrected some mistakes and incorporated some new features in the latest edition. We hope this program can provide researchers an easy instrument to develop further measuring methods that can be applied in plain radiograph.

## Implementation

Many reported methods used Fourier transformation to fasten the process in generating the radiographs from computed tomography data [15,16]. However, Fourier transformation may decrease the precision, which is the first priority in the measurement analysis on plain radiographs. On the other hand, ray tracing, which is popular in computer game, may be suitable for this transformation. Unfortunately, current built libraries only provide reflection images instead of transparent images that are needed in our analysis. Therefore we have to build up our whole software program before practical application in the plain radiographs.

Another problem is physics, i.e., once X-rays beam pass through the prosthesis, they then generate the image on the radiogram film. The grey scale on the radiogram film is determined by the amount of the X-ray passed that is dependent on the thickness of the metal in the pathway. Such condition follows Beer-Lambert law.

(1) Penetration = e^-*kbc*^

*k*: molar absorbability

*b*: path length

*c*: concentration

The parameter *k *is different among various metals and radiation energy (*kv *in X-ray). In real X-ray machine, the distribution of *kv *follows the rule of normal distribution, which is different among various X-ray machines. After calculating the amount of X-rays passed, we need another formula to transfer them to grey scale.

**Figure 2:**
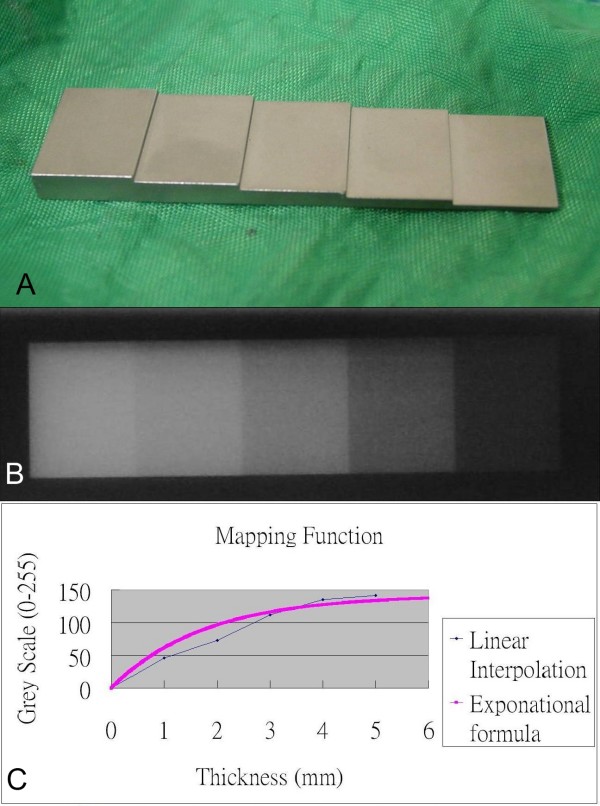
**(A)The phantom, which is made of titanium with thickness from 1 mm to 5 mm**. (B) The corresponding photodensity on the radiographs of the phantom is shown. (C)The mapping function (from thickness to grey scale). We fit the experiment points by an exponential function.

Because too many parameters needed to be controlled to build up the software program, we tried another solution. We first took X-ray (63 kv, 17 mas) on the step-wedge phantom, made of titanium, which has 5 steps with an increment of 1 mm thickness from 1 to 5 mm [Figure 2]. Such a step-wedge phantom film was scanned. The grey levels on this X-ray radiogram were mapped as the optical density values. We measured the optical density of each of the 5 wedges as well as that of the film background. The mapping function, from metal width to greyscale, is fit by exponential function. The mapping function is shown in Figure 2.

Our goal is to build a simulated total hip prosthesis. Virtually, femoral head equals to a ball.

(2) *x*^2^+*y*^2^+*z*^2^<*r*_*f*_^2^

(*x*, *y*, *z*) is the point of the simulated three-dimensional Cartesian coordinate system. *r*_*f *_is the radius of femoral head.

In our program, we make the ball move to simulate wearing of insert.

(3) (*x*-*d*_*x*_)^2^+(*y*-*d*_*y*_)^2^+(*z*-*d*_*z*_)^2^<*r*_*f*_^2^

*d*_*x*_, *d*_*y*_, *d*_*z *_are femoral head movement in three directions.

Virtually, acetabulum is composed of two balls and one plane.

(4) *x*^2^+*y*^2^+*z*^2^<*r*_*ao*_^2^

(5) *x*^2^+*y*^2^+*z*^2^>*r*_*io*_^2^

(6) *ax*+*by*+*cz*>0

*r*_*ao *_means radius of acetabulum's outer shell, *r*_*io *_means radius of acetabulum's inner shell, (*a*, *b*, *c*) means the normal vector of the acetabulum which can be derived from inclination and anteversion of acetabulum. Liaw et al. derived the following formula for this process.[17]

(6.1) (*a*, *b*, *c*) = (sin*φ *× cos*θ*, -cos*φ *× cos*θ*, sin*θ*)

Vector (*a*, *b*, *c*) means the normal vector of the acetabulum, *φ *means the inclination of acetabulum, *θ *means the anteversion of acetabulum, positive *θ *means anteversion, and negative *θ *means retroversion.

Theoretically, the X-ray source is set at (0,0,-*d*_*t*_). *d*_*t *_means tube distance (the X-ray tube to the acetabulum center). The points at film are (*x*_*f*_, *y*_*f*_, *d*_*f*_). (*x*_*f*_, *y*_*f*_) means point at film. *d*_*f *_means distance from film to the acetabulum center.

(7) (*x*, *y*, *z*) = (*t x*_*f*_, *t y*_*f*_, *t*(*d*_*f*_+*d*_*t*_)-*d*_*t*_)   0<*t *<1

The ray-tracing algorithm means calculating every ray from X-ray source to film. We used formula (7), simulating every X-ray from source to film. We then determined the total length the X-ray beam passed through femoral head by calculating the length between the two extreme solutions of formulas (3) and (7). Finally, we came out at the total length the X-ray beam passed through acetabulum by calculating length between the two extreme solutions of formulas (4), (5), (6), and (7). We use analytical mathematics for these calculations. The detailed process is illustrated in Appendix section.

In summary, we first built a mathematical model of acetabulum and femoral head, formulas (3) to (7). The real dimension data was adopted to generate the proper prosthesis figure. Then we calculated the total thickness of metal the X-ray beams passed by the ray-tracing algorithm, and then transformed these digital data to grey scale by mapping function. Finally, the gray scale generated for the prosthesis was shifted according to the aforementioned method. We could generate various radiographs according to the different parameters used.

The functional parameters in the *THR Simulator *include the following:

Thickness: refers to the thickness of the acetabulum shell.

Film size: refers to the X-ray film size it simulates.

Tube distance: refers to the distance from X-ray tube to the acetabulum center.

Film distance: refers to the distance from X-ray film to the acetabulum center.

Anteversion: refers to the version of the acetabular cup. The user can choose either version (anatomical, radiological, or operational) to simulate. Negative value means retroversion.

Abduction: refers to the abduction (or inclination) of the acetabular cup.

Upper movement: refers to the femoral head moving upward.

Medial movement: refers to the femoral head moving medially.

Posterior movement: refers to the femoral head moving posteriorly.

Picture size: refers to the size of picture file to simulate.

## Results and discussion

Under Windows platform and using Borland C++ Builder programming tool, we developed the *THR Simulator *shown in Figure 1. In the *THR Simulator*, the user can incorporate many parameters before generating a simulated radiograph, i.e., with different parameters adopted, we can generate different radiographs. These can be used for further analysis.

**Figure 3:**
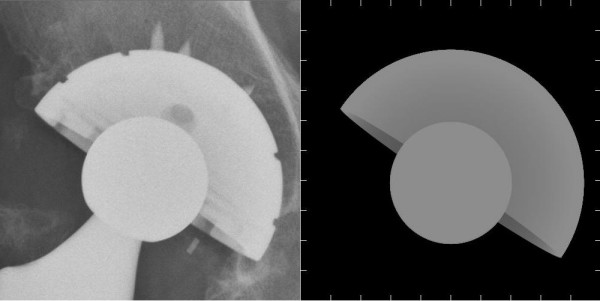
**The real radiograph is on the left, and the simulated one is on the right**. They show to be similar except some local features of the acetabulum and the bony noise.

As shown in Figures 1 and 3, we can obtain different simulated radiographs according to the parameters used. Thus we can demonstrate the function of such software, *Simulator*, in the application of the parameters.

Previously reported methods for measurement of acetabular cup orientation include mechanical simulator used for verifying insert wearing measuring methods [7], as well as other methods adopting Fourier transformation algorithm [15,16]. These methods have their own disadvantages. The mechanical simulator can be used to generate radiographs directly but has some inherent problems. The simulation process takes much time, about 50 seconds with Pentium III 500 MHz notebook. On the other hand, other methods using Fourier transformation algorithm can do it in real time [15,16] at the expense of lower precision. Thus we abandoned Fourier transformation algorithm and developed our own precise algorithm.

Our *THR Simulator *indeed improved the disadvantages of the above methods. The *THR Simulator *can incorporate several parameters for analysis at the same time. In practice, we can obtain the basic parameters from the prosthesis venders, i.e. shell diameter, shell thickness to generate simulated radiographs. Table 1 shows the parameters of total hip prostheses (U2, United Orthopedic Corp, Hsinchu, Taiwan).

**Table 1:** Parameters of total hip prostheses (U2, United Orthopedic Corporation, Hsinchu, Taiwan).

Acetabular shell diameter(mm)	Acetabular insert thickness (mm)	Femoral head diameter (mm)	Acetabular shell thickness (mm)
44	6.9	28	1.1
46	7.9	28	1.1
48	6.9	28	3.1
50	7.9	28	3.1
52	8.9	28	3.1
54	9.9	28	3.1
56	10.9	28	3.1
58	11.9	28	3.1
60	12.9	28	3.1
62	13.9	28	3.1

To make simulated radiographs more real, users can superimpose the synthesized radiographs onto real radiographs. We do not routinely recommend doing so because this action may make users misread patient's position and thus confuse the standardization process[17].

**Figure 4:**
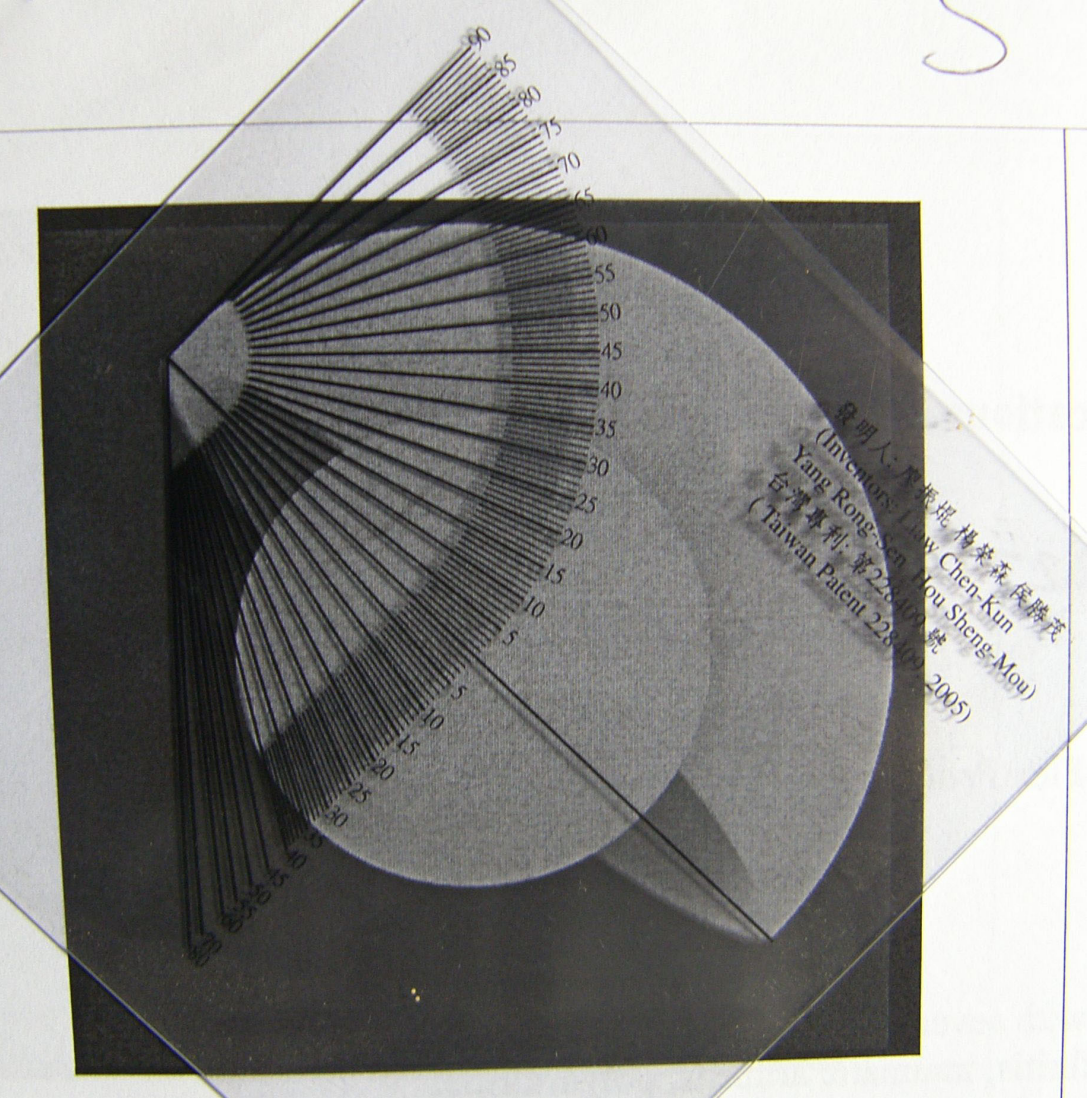
**The simulated radiograph is printed in paper and is measured with the previously published method**. We align the baseline of the protractor with the long axis of the ellipse, and then read the anteversion at the mid-point of the ellipse. Meanwhile we read the inclination at the horizontal line on the upper part of the protractor. In this case the anteversion is 9° and inclination is 45°.

The early edition of *THR Simulator *software has been used in two previous studies. One is to verify a new protractor in comparing with previous established mathematical method of measuring acetabulum[1]. We used the method in the earlier publication to simulate 45 radiographs of total hip arthroplasties with 15 different anteversions ranging from 15°–29°, and then verified the two methods of measuring anteversion of acetabular cup [1]. The mean errors of both measuring methods are within two degrees. Figure 4 shows how to measure the cup orientation with this invented method. The second study is to verify measuring insert wearing program. We use the earlier publication to simulate 64 radiographs with 2 different anteversion angles, 2 different abduction angles, 4 different superior wears, and 4 different medial wears. The errors are within 0.2 mm [18]. These results can also approve the precision of our *THR Simulator*. *THR Simulator *can provide researcher a new tool when developing their new device or new methods on measuring geometrical parameters of pelvis after total hip arthroplasty.

We use upper, medial, and posterior movements to indicate wearing of the three directions. Because femoral heads may not locate in the center of the acetabulum, the user should adjust these three vectors to fit every situation.

Our program can accommodate a three-dimensional wear vector by incorporating movements in the inferior-superior (upper), medial-lateral, and anterior-posterior directions. Inferior-superior and medial-lateral wears change the location of the femoral head relative to the cup while anterior-posterior wear changes the apparent size of the femoral head. However, the change in size is negligible.

Calculating wearing volume is another interesting issue. Kosak et al. has published a mathematical model to calculate it with the three wearing directions.[19]

## Conclusion

We designed new software *THR Simulator *that can generate radiographs after total hip arthroplasty. The strength is its accuracy and precision. The limitation is that it can not synthesize the details of the prosthesis and surrounding bone. We hope it can be used in future studies about measurements of geometrical parameters of pelvis after total hip arthroplasty.

## Availability and requirements

The software *THR Simulator *is attached [see Additional files 1, 2, 3]. It can be run in Microsoft Windows 98 and XP. The central processor and memory requirements are minimal. The three files should be located in the same directory in disc.

## Competing interests

The authors declare that they have no competing interests.

## Authors' contributions

TYW and RSY designed the study. TYW gathered the data. CKL, SMH and RSY analyzed the data. CSF wrote the initial drafts and CKL ensured the accuracy of the data and analysis.

## Appendix

Mathematical detail of calculating metal thickness

Equation (7) shows the line from X-ray source to the film.

We first change Formula (3) to Equation (3e).

(3e) (*x*-*d*_*x*_)^2^+(*y*-*d*_*y*_)^2^+(*z*-*d*_*z*_)^2 ^= *r*_*f*_^2^

Then we find the solution of *t *between Equations (7) and (3e). If there is no solution or only one solution, the thickness is zero, otherwise it means that the X-ray beam pass through it. From *t *we can calculate the real point using Formula (3), and then we can calculate the distance between the two points, which is the metal thickness the X-ray passed.

Similarly, we change Formula (4), (5), and (6) to Equations (4e), (5e), and (6e).

(4e) *x*^2^+*y*^2^+*z*^2 ^= *r*_*ao*_^2^

(5e) *x*^2^+*y*^2^+*z*^2 ^= *r*_*io*_^2^

(6e) *ax*+*by*+*cz *= 0

Then we find the solution of *t *between Equations (7) and (4e). If there is no solution or only one solution, the thickness is zero, otherwise it means that the X-ray beam pass through it. We keep the solutions of *t *in the solution set. Similarly, we find the solution of *t *from Equations (7) and (5e). Then we keep the solutions of *t *in the solution set if there are solutions.

We solve t from Equation (7) and Formula (6) and get the range of *t*, i.e. *t*>*rx *or *t*<*rx*, and *rx *is the solution of *t *from Equations (7) and (6e).

We exclude the solutions in the solution set outside the range of *t*. If the number of solutions in the solution set is odd, we append *rx *into the set.

Now we sort the solutions in the set, and pair the solutions with their neighborhood. We apply these paired *t *solutions to Equation (7) and get pairs of points.

Finally we add the distance of these pair points to we previously calculated distance intra-head. The sum is the total thickness the X-ray beam passed through the metal.

## Pre-publication history

The pre-publication history for this paper can be accessed here:



## Supplementary Material

Additional File 1 **THR Simulator. **The main program of our software. Click here for file

Additional File 2 **Borland dynamic library #1**. The dynamic library provided by Borland company. Click here for file

Additional File 3 **Borland dynamic library #2.** The dynamic library provided by Borland company. Click here for file
